# Disengaging polymerase: Terminating RNA polymerase II transcription in budding yeast^☆^

**DOI:** 10.1016/j.bbagrm.2012.10.003

**Published:** 2013-01

**Authors:** Hannah E. Mischo, Nick J. Proudfoot

**Affiliations:** a Cancer Research UK London Research Institute, Clare Hall Laboratories, Blanche Lane South Mimms, Herts EN6 3PS, UK; b Sir William Dunn School of Pathology, South Parks Rd, Oxford OX1 3RE, UK

**Keywords:** Eukaryotic RNA transcription, RNA polymerase II, 3′ end processing, Transcription termination

## Abstract

Termination of transcription by RNA polymerase II requires two distinct processes: The formation of a defined 3′ end of the transcribed RNA, as well as the disengagement of RNA polymerase from its DNA template. Both processes are intimately connected and equally pivotal in the process of functional messenger RNA production. However, research in recent years has elaborated how both processes can additionally be employed to control gene expression in qualitative and quantitative ways. This review embraces these new findings and attempts to paint a broader picture of how this final step in the transcription cycle is of critical importance to many aspects of gene regulation. This article is part of a Special Issue entitled: RNA polymerase II Transcript Elongation.

## Highlights

► Pol II transcription termination and 3′ end processing are different processes. ► Termination and 3′ end processing are intimately connected and influence each other. ► Pol II transcription termination employs two non-mutually exclusive pathways. ► Many cues transmitted to Pol II influence its choice of termination pathway. ► Termination choice is affected by external stimuli, which controls gene expression.

## 1 Introduction

At the end of a transcription cycle RNA polymerase disengages from the template DNA and releases its transcript. Initial scrutiny of protein-coding genes transcribed by RNA Polymerase II (Pol II), suggested that this was a fairly discrete process; upon recognition of a Pol II termination signal, the transcribed nascent RNA is processed and Pol II released from the template [1,2]. However, discoveries of the past few years have revealed an unanticipated complexity and connectivity between transcription termination, RNA 3′ end processing and the transcription cycle.

In this review we will focus on the connected mechanisms of transcription termination and RNA 3′ end rocessing. We will also provide selected examples of how transcription termination and RNA 3′ end processing feed into the larger context of gene expression. Several recent excellent reviews have covered the mechanism of transcription termination and RNA 3′ end processing in mutually complementary ways [3–5]. Although some redundancy is unavoidable, the pace at which new findings broaden our mechanistic and biological understanding of transcription termination per se and gene expression in general merits another attempt to summarise, generalise and put new facts into context; even so, we anticipate that future findings will make some of our current generalisations seem precocious. The mechanism of transcription termination appears to be fairly conserved in the different kingdoms, but its molecular analysis in yeast preceded in many cases that of mammals. We will therefore largely confine our review to the situation encountered in *S. cerevisiae*, providing *H. sapiens* nomenclature, where applicable, in brackets. We refer the reader to very detailed reviews covering additional mammalian specificities [3–5].

## 2 Genome wide chromatin structure as a transcriptional stage

**Fig. 1:**
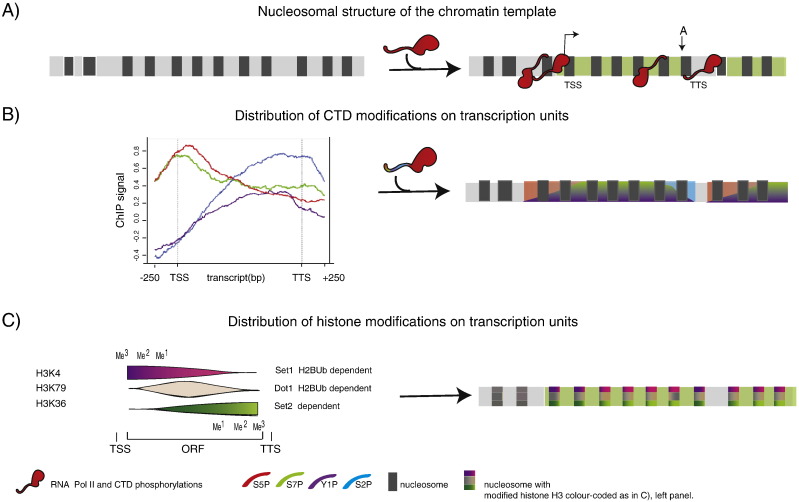
Schematic drawing of chromatin environment associated with Pol II transcription. A) Nucleosome free regions (left panel) determine, where Pol II finds access to DNA, establishing transcription units (coloured in green, right panel). B) Co-transcriptional phosphorylation of the Rpb1 CTD influences which proteins can bind to the polymerase. Left panel: CTD phosphorylation.The average is taken over 339 yeast genes of medium length (1,238 ± 300 nt). Only those genes belonging to the 50% most highly expressed genes and that were at least 200 nucleotides away from neighboring genes are considered. Based on results from Michael Lidschreiber and Patrick Cramer [59,60]. The right panel shows the prevalent phosphorylation form likely to be sensed by Pol II based on the left panel colour coding. C) Some of the proteins bound to the CTD will affect histone modifications. Colour-coded histone modifications characterize distinct regions of transcription units (adapted from [228]).

The advancement of genome wide techniques has not only allowed the generalisation of findings from well-studied, isolated examples, but has also helped uncover hitherto unnoticed biological phenomena. Whereas classically the three eukaryotic RNA polymerases (Pol I, II and III) had clearly separate responsibilities in the gene expression repertoire, deep sequencing techniques have uncovered many new transcription units for both Pol II and III that often give rise to short lived or regulatory non-coding RNA [6–8]. Especially Pol II has a remarkable propensity to engage with DNA wherever the chromatin template is depleted of nucleosomes (Nucleosome Free or Depleted Region, NFR or NDR) [9–12]. Pol II chromatin engagement is mediated by the formation of a pre-initiation-complex (PIC) that will direct the initial stages of transcription [13]. Although it was well known that perturbation of nucleosome positioning can result in isolated cryptic/unscheduled transcription events [14,15], it is now apparent that even in the unperturbed chromatin context Pol II engages with PICs formed on DNA wherever they find access (Fig. 1A) [16]. In many cases this results in un-productive transcriptional activity, whilst in others it adds to a large repertoire of non-coding, short lived or stable, functional or non-functional transcripts [17,18]. The biological role of these transcription events and their resulting transcripts is fascinating where understood but in many other cases awaits further scrutiny [e.g. 8,19,20].

In addition to allowing for unscheduled transcriptional events, perturbation of nucleosome positioning will severely affect the ability of Pol II to progress into productive elongation or even to terminate transcription [21,22]. To what extent these observations can be generalized and are a direct consequence of altered nucleosome positioning, remains to be tested. However several studies suggest that as with transcription start sites (TSS), some transcription termination sites (TTS) acquire specific nucleosome positioning patterns that can be correlated with distinct polymerase occupancy and expression levels [23–26]. As discussed by Schmolle and Workmann in this issue, similar to nucleosomal positioning, nucleosomal histone modifications can also be correlated with transcriptional activity as they can drive or suppress transcription [27–30]. A growing network of physical and genetic interactions is observed between transcription elongation factors and chromatin remodelers. In many cases these are orchestrated by histone modifications, indicating that chromatin structure provides an important overlying, yet plastic, control level to Pol II transcriptional activity. The fine-control of this process is far from understood (Fig. 1C) [31–36].

In our subsequent discussion of the mechanism of transcription termination we will therefore distinguish between controlled or scheduled transcription termination and unscheduled transcription termination. The latter describes a large hub of partially ill-defined events ranging from termination of abortive transcripts to premature termination of polymerases that encounter obstacles during transcription along a gene. In making this distinction, we would like to emphasize that as for the newly identified “short” (or longer) non-coding transcripts, even in a regular chromosomal context transcription processivity is often low. As a consequence, prematurely terminated transcripts can outnumber correctly terminated ones [37–41]. As scheduled transcript termination feeds into a production line that results in a mature coding or non-coding RNA, non-scheduled transcript termination is equally coupled to RNA turnover mechanisms that act to restrict RNA synthesis and polymerase function. The complexity of the various transcriptional quality control mechanisms, as discussed by Jensen and colleagues in this issue, further emphasizes how critical the control of unrestricted transcription is to the cell.

## 3 Messenger RNA synthesis by RNA polymerase II

All three eukaryotic RNA polymerases have the ability to cleave RNA within their active sites. However *in vitro* studies suggest that this cleavage process is only sufficient to lead to Pol I and Pol III release at designated sites [42,43]. In contrast, Pol II transcription termination and 3′ end processing requires a set of different protein complexes to bind to the polymerase as well as to specific sequences in the nascent RNA. Changes in composition or posttranslational modifications of these factors greatly increase their capacity to regulate transcript processing and transcription termination. In most protein coding genes, a poly(A) signal (PAS) doubles as a 3′ end processing and TTS, so that subsequent polyadenylation will stabilize the RNA. However many other Pol II transcripts are non-polyadenylated and are terminated at different TTS. To prevent degradation of these RNA, specific RNA–protein interactions are required. Discoveries in recent years have shed some light on the extensive repertoire of TTS available for Pol II and how these differentially affect the fate of the RNA transcribed [4,5,44].

### 3.1 Processing of transcripts from coding genes

**Fig. 2:**
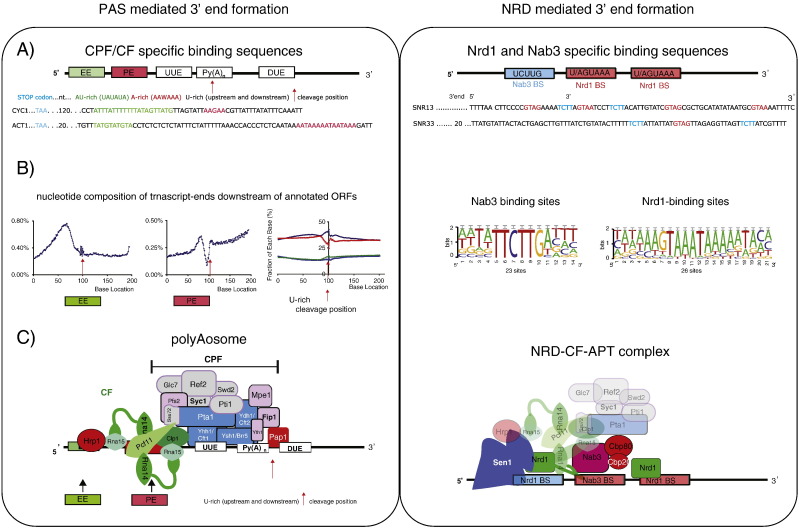
3′ end formation signals in yeast. A) Left: CPF/CF specific binding sites are characterised by the AU-rich efficiency element (EE), the A-rich positioning element (PE) and U-rich regions surrounding the cleavage site (upper). Polyadenylation signals of two mRNAs are shown to exemplify the low conservation of the different sequence elements (lower). Right: NRD-dependent terminators, characteristically have 1 to several Nrd1 or Nab3 binding sites, where Nrd1 binding sites have the tendency to cluster, and Nab3 binding sites can occur in isolation (upper). Two RNAs with NRD dependent terminators are shown to exemplify the variation in spacing and composition of Nrd1 and Nab3 binding sites in the RNA sequence (lower). B) Left: Genome wide analysis shows the percent-distribution of the EE (TARYTA) and PE (AAWAAA) elements with respect to the cleavage site (left and middle), as well as a preference for AT richness 40–50 nt upstream of cleavage positions (right) [50]. Due to little conservation and lack of motifs in the sequences surrounding mapped cleavage sites of polyadenylated messages, the nucleotide distribution is depicted as percentages and TGCA are depicted in dark blue, blue, green or red respectively. Right: Nrd1 (left hand side) and Nab3 (right hand side) consensus sequences as identified by genetic screening (Porrua-Fuerte et al. 2012). C) Suggested stoichiometry and organisation of the CPF/CF complex on mRNAs (left) and the NRD-CFI-APT complex on non-coding RNAs (right). Sub-complexes are colour-coded: blue, pink, grey, CPF with the subcomplexes APT (grey) and CFII (blue). Green and red (Hrp1) are CFI. The NRD-CFI-APT complex has not been biochemically purified, but been inferred from genetic studies.

Where a PAS serves as a TTS, the transcript is processed by endonucleolytic cleavage and subsequently protected from 3′ end degradation by addition of a poly(A) tail of about 70 nt (200 nt in mammals) [45]. In yeast, this task is performed by over 20 proteins that are organized in several sub-complexes (Fig. 2C, left). The mammalian “polyAosome” is even more complex being composed of some 80 polypeptides which may reflect the tight interconnectivity of 3′ end processing with other RNA processing events (Section 6) [1,46,47].

In contrast to polyadenylation, termination of transcription occurs where Pol II is released from the DNA template over a range of about 200 (yeast) to 1500 (humans) nt downstream of the PAS [48,49].

The PAS of humans consists of a highly conserved hexanucleotide sequence, AAUAAA, in addition to other less conserved sequence recognition elements, whereas *S. cerevisiae* PAS are more degenerate so that their consensus is sometimes difficult to recognize (Fig. 2A, left) [1,50,51]. For most *S. cerevisiae* genes, they consist of an adenine-rich efficiency element (EE, TAYRTA, with Y being any pyrimidine, R being any purine and W being adenine (A) or thymine (T)), an A-rich positioning element (PE, AAWAAA), typically located 30 nt upstream of the cleavage position, as well as an uridine-rich element spanning the cleavage position and site of poly(A) addition (Fig. 2B, left) [52–56]. The moment a poly(A) signal is transcribed by elongating Pol II, two multimeric complexes, CPF (cleavage and polyadenylation factor) and CF (cleavage factor I A and B) bind to these elements in the nascent pre-mRNA (Fig. 2C, left) [57]. Various factors in these complexes also bind to the Pol II C-terminal domain (CTD) of Rpb1 subunit during transcription. CTD is a flexible C-terminal extension harbouring 26 (52 in humans) hepta-peptide (consensus Y_1_SPTSPS) repeats that are dynamically phosphorylated during the transcription cycle [58–62]. By binding of CPF/CF components to the CTD they can be enriched at a particular stage during the transcription of a gene [58,60]. The concentration of specific RNA binding proteins on the CTD facilitates the recognition of specific RNA sequences and thereby greatly enhances the efficiency of the cleavage and polyadenylation reaction [63]. Amongst the various CPF/CF proteins that interact with phosphorylated CTD, a component of CFI called Pcf11 contains a CTD interacting domain (CID) that enhances its binding to gene 3′ ends, at which point CTD carries stimulatory serine 2 phosphorylation (Ser2P) and lacks inhibitory Ser5P or Tyr1P [58–60,64] (Fig. 1B).

However, even if yeast cells are momentarily induced to express Pol II devoid of its essential CTD, some 3′ end processing can still be observed, arguing that interaction with CTD is not an absolute prerequisite for 3′ end processing [63].

In yeast, the RNA cleavage site is defined by binding of CF IA component Rna15 to the PE and a loosely associated protein Hrp1 (CF IB) to the EE [56,65–69]. CF IA furthermore contains Rna14, Pcf11 and Clp1, which are all essential for the 3′ end processing and termination reactions. They also function to form a scaffold to tether the catalytic CPF to the poly(A) signal [70–72]. Within this scaffold, two Rna14 and Rna15 heterodimers associate dynamically with Clp1 and Pcf11 [46,73], whereas both sub-complexes are brought into physical contact through interaction of Clp1 to several CPF components [72,74]. Clp1 and Pcf11 will be discussed further below (Section 3.2), as both have essential, yet not completely understood functions in both 3′ end processing as well as in the termination of transcription.

The enzymatic activities for cleavage and polyadenylation are concentrated in the 15 subunit CPF complex that binds further downstream over the site of 3′ end cleavage (Fig. 2C, left, blue, grey, pink colouring) [75]. In particular, Yhh1 and Ydh1, bridge the poly(A) signal with the phosphorylated CTD [76,77]. They are also in direct contact with other CPF and CF subunits, and together with Pta1 may form a central scaffold [78]. Of the two proteins that contain a metal–ion endonuclease fold (βCASP-family) Ydh1 (CPSF-100) and Ysh1 (CPSF-73), only Ysh1 cross links to the cleavage site and contains critical amino acids required for endonucleolytic function [79–82]. Through interaction of Yth1 with Fip1, or directly with Pta1, Pap1 the poly(A) polymerase is tethered to the complex [83,84]. It is possible that Yth1 coordinates rearrangements of the complex before the cleaved transcript is polyadenylated by Pap1, a reaction which in yeast is controlled by Nab2 and Pab1 that restrict poly(A) tail synthesis to an average length of 70 nt. In mammals the non homologous PABII cooperatively stimulates the synthesis of a 200 nt long poly(A) tail [45,85–91]. Since 3′ end processing is a final step in mRNA maturation, the polyadenylated transcript is then ready to leave the nucleus.

About half of the cellular pool of core CPF associates with six additional polypeptides through interaction with Pta1 [92]. This APT complex (associated with Pta1) contains several proteins that have additional functions at early transcriptional stages, such as Ssu72, the CTD Ser5P phosphatase activity and Swd2, also a component of the Set1 methyl transferase complex COMPASS (Fig. 2C, left, grey colouring) [93,94]. Although the function of APT in 3′ end processing is not completely understood, available evidence suggests that it is required for fine-tuning of the 3′ end processing reaction (Section 4).

The degenerate character of poly(A) signals in yeast has two consequences. First, a slight perturbation in mRNA packaging or altered CTD binding of the 3′ end processing factors, may result in changes of 3′ end selection through usage of more diverse poly(A) signals [50]. For instance, an increase of mRNA packaging caused by Npl3 over-expression can hinder CFI-component Hrp1 recruitment and recognition of weak cryptic poly(A) sites [95–97]. On the other hand, if the local concentration of 3′ end processing factors that interact with the Ser2P CTD is reduced, cleavage and polyadenylation still occurs, albeit slower and preferentially at strong poly(A) signals [98–100]. These studies in combination imply that while *in vitro* uncoupled 3′ end processing can occur, the efficiency of this process is greatly enhanced when coupled to regulated mRNA packaging and association with Ser2P CTD [101].

### 3.2 Transcription termination of protein coding genes

Transcription termination occurs up to several hundred bases downstream of the 3′ end processing site even though both processes are intimately linked and mutation of many 3′ end processing factors results in transcription termination defects [48,76,93,98,102–105]. Similarly, when a degenerate poly(A) signal is mutated this will fail to stimulate termination *in vivo* even though *in vitro* the surrounding sequences may stimulate some residual cleavage activity [68].

After many years of debate on exactly how Pol II is disengaged from the template at a poly(A) signal, it seems plausible that two key aspects influence Pol II destabilisation at poly(A) signals; the speed of the Pol II elongation complex (EC), as well as the stability of the RNA:DNA hybrid within the active centre of the polymerase body. Whereas the RNA:DNA hybrid is probably the main determinant of Pol II EC stability, modulating the EC speed will affect how much time other proteins have to act on it. In a plethora of examples Pol II was observed to pause downstream of poly(A) signals *in vivo* and *in vitro* and these examples have now been generalised by genome wide studies showing that polymerase speed decreases after many poly(A) signals [23,40,106–110]. Such transcription deceleration in the vicinity of a poly(A) signal can be correlated with a dramatic change in factors associating with and affecting Pol II transcriptional speed [60,111]. In some particular cases, proteins binding to specific DNA sequences can also form roadblocks and hinder Pol II progression [112,113].

Several models for how the RNA:DNA hybrid within the polymerase body could be destabilized have been suggested: Firstly, a processive protein was suggested to engage with the nascent RNA, follow and destabilize a paused Pol II by reducing the RNA:DNA hybrid length in its active site. Secondly if a protein is associated with Pol II close to the RNA exit channel, this could invade the polymerase active site and unwind the hybrid without directly possessing processivity itself being processive [105,114,115]. It seems that *E. coli* employs both these mechanisms as manifested by the hexameric ring-shaped transcription termination factor Rho. This factor associates with transcribing polymerases and appears to thread the RNA through its body, until the polymerase undergoes a conformational change that disengages RNA polymerase from the DNA template [116]. Consistent with the first model, Rho is also able to displace paused yeast Pol II in an ATP-dependent *in vitro* reaction, whereas it has no effect on termination of paused Pol I or III [112]. Since no close homologue of Rho exists in eukaryotes, the major nuclear 5′-3′ exonuclease Rat1 (Xrn2) [117,118], was tested for its ability to disengage Pol II. Encouragingly its mutation does display a mild termination defect *in vivo,* even though the isolated enzyme fails to dismantle Pol II from DNA templates in *in vitro* reactions [119–121]. An explanation for this weak phenotype could either lie in the nature of the experimental system or the fact that the effect of Rat1 mutation is hidden by redundant factors. A cytoplasmic homologue of Rat 1 (Xrn1, 28–39% identity in the catalytic domains) can, when artificially localized to the nucleus, complement Rat1 mediated RNA degradation, but not termination — as it is not recruited to 3′ ends of genes [122]. Redundancy with Xrn1 can't therefore account for the weak phenotype of Rat1 mutation [123,124]. The search for potential regulatory subunits of a Rat1 complex is ongoing: Rat1 co-purifies with two proteins, Rai1 (Rat1 interacting protein) and Rtt103 [120]. Both localize to 3′ ends of genes, but their deletion does not result in drastic transcription termination defects at poly(A) signals. In conclusion it seems that although the pyrophosphatase Rai1 aids and stimulates Rat1 exonuclease activity *in vitro* [125], *in vivo* Rat1 acts in combination with Rai1 to degrade rRNAs that are polyadenylated as part of a quality control mechanism involving the nuclear exosome component Rrp6 [126,127]. Similarly, neither Rtt103 interaction with Ser2P CTD, nor CTD Ser2P at 3′ ends of genes, are responsible for Rat1 recruitment [120]. As discussed below (Section 4.2), these combined observations question the dominance of Rat1 in PAS associated termination and argue for the existence of additional termination factors.

Because Rat1 prefers to engage with 5′ monophosphorylated RNA ends, a possibly heightened role for Rat1 in termination came from the observation that human CF IIm component ClpI (hClpI) has a 5′OH RNA kinase activity [128–131]. However, this activity is not conserved in yeast cells and the hClp1 ATP-binding homologous domain is pivotal for interaction with Pcf11 [74]. It therefore seems that hClp1 either is evolutionary different or fulfils additional functions outside the CF IIm [72,132]. Pcf11 has been shown to destabilize transcription elongation complexes in several organisms [105,114,115]. The underlying mechanism of this activity is not yet completely understood, but requires Pcf11 to bind both the Ser2P CTD and the RNA. Since CTD interaction and 3′ end processing activity lie within separate domains of Pcf11, and other factors within CF IA, such as Rna14, also bind RNA and phosphorylated CTD, it will be interesting to further dissect the action of Pcf11 [69,103]. In addition, mutation of Pcf11 results in stabilisation of the 3′ nascent RNA, possibly because in these mutants Rat1 association with 3′ ends of genes is diminished [122]. Similarly, mutation of Rat1 results in diminished association of several 3′ end processing factors with 3′ ends of genes, although *in vitro* 3′ end processing itself is not affected [120,122]. These combined observations once more indicate that for transcription termination to occur at a PAS, multiple activities have to concentrate in a coordinated fashion. However, how much PAS associated Pol II termination depends on the RNA sequence, Pol II modifications or chromatin signatures still remains incompletely answered.

## 4 Non-coding RNAs transcribed by RNA polymerase II

In addition to polyadenylated transcripts, Pol II transcribes several classes of non-coding RNA, such as small nuclear RNA (snRNA), small nucleolar RNA (snoRNA), stable unannotated transcripts (SUTs), cryptic unstable transcripts (CUTs), meiotic unannotated transcripts (MUTs) and Xrn1 stabilized transcripts (XUTs); the short RNAs found at mammalian TSS or promoter upstream transcripts (PROMPTs) [18,133–136]. These RNAs differ greatly in abundance and function, but their transcription termination and 3′ end processing, where understood, appears to fall into one of two categories: Some SUTs and XUTs probably rely on the above described mechanism employed for PAS dependent termination [38,134,137]. For others, such as snoRNA, snRNA and CUTs, transcript processing and termination depend on components of the CPF, CFI and the NRD complex (aka Nrd1-Nab3-Sen1-dependent pathway) [133,136,138]. How these protein-complexes act together to terminate non-polyadenylated RNAs remains incompletely described, but similar to poly(A) dependent termination, specific RNA sequences are recognized by proteins within NRD (Section 4.2 and Fig. 2, right) [139]. NRD dependent termination is associated with very different transcriptional outcomes: It can result in the generation of stable and highly abundant (10^5^–10^6^ copies per cell) snRNAs of the spliceosome, and snoRNAs, involved in rRNA biogenesis. In contrast, CUTs are transcribed at varying levels, presumably from accessible chromatin acting as “adventitious” and bi-directional promoters [9,18,26]. Their termination by NRD is coupled with their rapid degradation under most biological conditions [133,140].

### 4.1 Processing of stable non-polyadenylated transcripts

Mammalian promoters of either polyadenylated or non-polyadenylated RNA genes are clearly distinguishable [141,142]. Consequently, mammalian sn- and snoRNA transcription shows various specialised characteristics that differentiate its transcription from polyadenylated RNA transcription, as recently reviewed [3,143]. In contrast, yeast promoters of coding and non-coding genes show no obvious differences so that the decision for which particular mode of termination is to be employed may be made at later stages of the transcription cycle.

During transcription of snRNA and snoRNA, these transcripts associate with specific proteins to form snoRNA particles (snoRNP, formed by H/ACA or C/D-box proteins) or snRNPs (formed by the Sm-class proteins) [144–147]. Association with these proteins stabilizes the three-dimensional structure of the RNA and ensures its stability, but is also required for proper 3′ end formation and transcription termination [148,149]. Whilst many snoRNA in mammalian cells are processed from polycistronic or intronic transcripts, the majority of *S. cerevisiae* snRNA and snoRNA are transcribed from independent TATA box containing promoters (51 of 76 snoRNA genes encoded in *S. cerevisiae*). The 5′ NFR of many of these promoters is bound and demarcated by Tbf1, a protein known to prevent heterochromatin spreading at telomeres [113]. In about half of these sites, Tbf1 is joined by Reb1, a factor also required for Pol I transcription [150]. As Tbf1 binding strongly stimulates snoRNA expression, it is possible that Tbf1 binding establishes directionality of these highly transcribed promoters by blocking divergent transcription and thus constitutes another termination event.

The termination mechanism of snoRNA genes, although employing some common factors, differs markedly in several key aspects from PAS mediated termination. First, the main RNA processing step involves exonucleolytic transcript trimming from both ends; 5′ to 3′ by the exonucleases Rat1 (Xrn2) or Xrn1 and 3′ to 5′ by the nuclear exosome, a protein complex with two 3′ to 5′ exonuclease activities [151–153]. The exosome engages with a 3′ OH of the snoRNA that can be generated by either of three ways: in the case of polycistronic RNA, the endonuclease Rnt1 cleaves between the several snoRNAs [154,155]. In contrast, for monocistronic RNA either transcript cleavage by CPF or release from the polymerase through transcription termination can both potentially provide a 3′OH end [156,157].

Second, snoRNA termination and processing relies on a different use of the 3′ end processing machinery. In addition to CTD Ser2P (and the responsible kinase Ctk1), they require all components of CF IA (Rna15, Rna14, Clp1, Pcf11), but only those of the APT sub-complex of CPF (Glc7, Swd2, Pti1, Pta1, Ref2, Ssu72) [72,78,92–94,138,156,158–160]. This requirement may indicate that transcript cleavage is not a prerequisite for RNA 3′ end trimming and that instead all the functions of CPF required for proper snoRNA processing are concentrated in APT. Several observations indicate that APT function lies in fine tuning and possibly the suppression of CPF activity: if co-purified with Pti1 or Ref2, CPF loses its ability to stimulate Pap1 dependent polyadenylation [158]. An N-terminal fragment of Pta1, a major scaffold of APT and CPF, inhibits cleavage and polyadenylation by CPF [78]. Similarly, deletion of the Pta1 N-terminal 75 amino acids, leads to degradation of Ssu72 and consequent global increase of Ser5P CTD, thereby indirectly inhibiting cleavage and polyadenylation [78]. Finally, *in vitro* the requirement of Ysh1 for transcript cleavage and polyadenylation can be suppressed by deletion of Syc1 (similar to Ysh1 c-terminal domain). It has therefore been proposed that Syc1 competes with Ysh1 for either inhibitory or stimulatory interactions [82].

In contrast, if adventitious polyadenylation of a snoRNA occurs, this interrupts snoRNP interactions and so may direct the RNA for degradation [156]. Since 3′ end formation and polyadenylation is directly coupled to mRNA export, the suppression of snoRNA polyadenylation may serve to ensure their nuclear retention. Overall, these unique aspects of snoRNA 3′ end formation imply that CPF action can be taylored to its substrate by the presence of APT. This variability of CPF is further exemplified by the intricate 3′ end processing of non-polyadenylated mammalian histone pre-mRNA in which (in contrast to yeast histone mRNAs) endonucleolytic cleavage is controlled by a different combination of factors as discussed in recent reviews [161–163].

### 4.2 Transcription termination of stable non-polyadenylated transcripts

snoRNA termination sites are defined by arrays of two oligo-nucleotide motifs UCUU*G* and *(U/A)*GUA(A/G) (Porrua-Fuerte et al. 2012, *in press*) [156,164,165] (Fig. 2A, right panel). These elements are bound by two essential proteins Nrd1 and Nab3 that co-purify in a complex with Pol II and the essential super family I helicase Sen1. These factors also copurify with components of the nuclear exosome, the cap binding proteins Cbc20 and Cbc80, as well as with Glc7, an APT component and phosphatase that also interacts with Sen1 [94,166] (Fig. 2C, right panel). Nrd1 (nuclear pre-mRNA down regulation 1, or the human SCAF8 (SR-like CTD-associated factor 8)) possesses a central RNA recognition motif (RRM), and an N-terminal CTD interacting domain (CID), which establishes interaction with Ser5P and Ser2P CTD [59,167–169]. Nrd1 interacts physically and genetically with Nab3 (nuclear poly(A) RNA binding 3), similarly an RRM and a potential C-terminal arginine/glutamine multimerisation domain containing protein [170,171]. Cooperative binding of multiple Nab3–Nrd1 heterodimers to several sequential copies of Nrd1 (GUAA/G) and Nab3 (UCUUG) binding sites as contained in snoRNA terminators elicits termination coupled to exosome processing [139,164,170]. However, although on a random basis these tetra-and penta motifs should occur every 256 to 1024 nt, they are depleted from coding regions. This implies a negative selection process that prevents both transcription termination within ORFs as well as premature termination of translation at stop-codons contained within the NRD binding sites (UAA and UAG) [6] (Porrua-Fuerte et al. 2012, *in press*). In contrast, Nrd1 and Nab3 binding site sequences are more prevalent in the AT-rich intergenic regions of short or long genes, and are also found in some promoter proximal regions, ORF antisense orientations, as well as tRNA elements. This suggests their involvement in gene expression regulation of a wide spectrum of transcripts [172]. However, only in combination with further AU-rich elements and if Nrd1 binds to a permissive Ser5P CTD will these sequences result in NRD-dependent termination (Section 5) [173] (Porrua-Fuerte et al. 2012, in press).

Mutation of the third essential gene within the NRD complex, Sen1 (Senataxin), causes the most severe and pleiotropic phenotype, affecting termination of both Pol I and Pol II transcription [94,138,166,168,174–183]. The large protein surface of Sen1 affords interaction with many different proteins and may thus be pivotal in bridging NRD processing and transcription termination functions [184]. For example, through interaction with Glc7, Sen1 not only helps the recruitment of CPF to snoRNA terminators, but may also enable Glc7 to dephosphorylate Sen1 and possibly Nrd1 [94,185,186]. As Sen1 displays the only enzymatic activity in this complex, it has been suggested that it is required for polymerase release. Although the mechanistic details of Sen1's role in transcription termination are as yet incompletely understood, it may act similarly to the Rat1 exonuclease as a Rho-like transcriptional torpedo (Section 3.2; O. Porrua-Fuerte, D. Libri, S. Buratowski and H. Mischo unpublished results). Engaging with RNA in the vicinity of NRD binding sites, it could chase and bind Ser2P Pol II, acting as a wedge between the RNA and the Pol II active site [185]. In support of this hypothesis, Sen1 has been shown to cooperate with Rat1-dependent termination in some instances [180]. However, as will be discussed below (Section 5), it is presently unclear to what extent this represents overlapping or cooperative functions of Sen1 and Rat1 or of the two termination mechanisms so far described [182].

Alternatively, Sen1 has been suggested to prevent hybridisation of the nascent RNA to underwound transcribed DNA which results in stable RNA–DNA structures [181]. As RNA–DNA hybrids generally interfere with Pol II processivity [40,187], they may be required to slow down Pol II in termination regions or allow access of other factors such as Nrd1/Nab3 or Pcf11. Such a model would suggest a targeted control of Sen1 function in termination regions, possibly through post-translational modifications such as dephosphorylation by Glc7 [94]. This becomes particularly appealing in the light of recent genome wide cross-linking studies which show that Sen1 is associated with the 3′ ends of many mRNAs and generally associated with transcribed gene regions [8,165,185]. Reinforcing previous studies, the Sen1 genome wide cross-linking pattern may therefore in addition to its role at NRD-dependent genes suggest a general function for Sen1 in the transcriptional termination of protein coding genes [165,172,180,182,185]. Indeed the human homologue of Sen1, Senataxin has been shown to play a direct role in mammalian Pol II termination. In some protein coding genes it resolves RNA–DNA hybrids to allow access of Xrn2 to the Pol II associated transcript [178]. It remains to be established whether Sen1 plays a similar role or alternatively, whether Senataxin may have evolved a discrete function from Nrd1 and Nab3 human homologues. Senataxin mutations show a pathologic phenotype that appears to be based on pleiotropic transcription processing and termination defects [179,188,189]. However, these phenotypes are only manifest in the cerebellum. The causative Senataxin mutation in the neurodegenerative disorders AOAII (ataxia ocular apraxia type II) and ALS4 (Amyelotropic lateral sclerosis 4) results in degeneration of the cerebellum and consequent loss of muscle function [190,191].

### 4.3 Transcription processing and termination of other non-polyadenylated transcripts

As with snoRNA and mRNA, transcription termination of the plethora of other RNA Pol II transcripts appears to be tightly linked to their processing. Although the growing list of such transcripts renders this discussion inevitably incomplete, we will attempt to summarize current emerging principles. Transcription termination of CUTs also depends on the NRD complex. However, lack of stable interactions of these RNA with proteins, ensues their immediate degradation through the NRD associated nuclear exosome [133,192,193]. Before they are recognized as substrates by the exosome, the terminated CUTs are in most studied cases oligoadenylated by a non processive poly(A) polymerase complex containing either the catalytic Trf4 or Trf5, as well as the regulatory Air1 and Air2 (aka the TRAMP complex) [194,133,140]. Similarly, mammalian PROMPTs are stabilized by siRNA mediated knockdown of the nuclear exosome, even though they may follow normal mRNA processing pathways [195,196]. The majority of the stable SUTs appear to be processed by the regular CPF/polyadenylation pathway, exported and finally degraded by the cytoplasmic nonsense mediated RNA decay pathway [137].

## 5 Redundancy and failsafe termination

In the above discussion we have reviewed the two major termination pathways, PAS and NRD dependent, as well as the different coupled transcript processing strategies available to Pol II. Even though this distinction may be useful for the dissection of the molecular mechanisms involved, there is little evidence *in vivo* that initiating *S. cerevisiae* Pol II receives any pre-determined cues in favour of one or another transcription termination pathway. In contrast it seems that Pol II behaviour is dynamically adapted in an opportunistic response to the factors influencing it. For example, nucleosome positioning determines chromatin accessibility to Pol II albeit not its directionality. An initial block to transcription can only be overcome if various positive transcription signals in combination exceed a threshold. By overcoming this threshold, transcription is selectively favoured in one direction. Transcription only proceeds throughout the body of the gene if obstacles do not hamper processivity. Equally, nucleosomes, removed by the passage of elongating Pol II, have to be rapidly re-assembled behind the transcribing polymerase. Pol II release only results in the production of a functional mRNP if a battalion of processing events are correctly coordinated in a timely manner.

When polymerase engages with the DNA template, transcription can be directed in both directions by PICs [9,13,18]. At highly transcribed genes, such as snoRNA genes, bi-directionality may require an active mechanism of termination in the non-favoured direction [113]. On the other hand, if not all factors required for processive elongation are present at a promoter, Pol II will quickly terminate. Nrd1 and Nab3 localize to the 5′ regions of many protein-coding genes and could provide one way to achieve early termination [165]. As Ser5P is still high in promoter-proximal regions, Nrd1/Nab3 RNA sequences can be recognized and used for termination and coupled transcript degradation. Importantly, the APT sub-complex, which is specifically required for NRD-dependent termination also localizes to the 5′ end of several ORFs [58,92,98,111]. In a growing number of gene examples (*NRD1, HRP1, PCF11, RPB10, MUD1, URA2, URA8, IMD2, IMD3, HPT1 , GUA1, ADE17, SER3, SRG1, CLN3, SMK1*) this termination mechanism is exploited for active transcript control in response to changing growth conditions [20,165,197,198]. Reminiscent of bacterial operon systems, two negative feedback loops for metabolic pathways rely on inhibition of the NRD pathway; low GTP-concentration results in increased expression of *IMD2* and *IMD3*, key regulators in the purine nucleotide biosynthesis pathway, whereas low nutrient concentrations will increase transcription of *SER3*, the 3-phosphoglycerate dehydrogenase [199–201]. Intriguingly, mutations in components of the NRD pathway also increase expression of Nrd1 itself, CF IB (aka *HRP1*), Pcf11 and the U1 snRNA binding protein A1 (*MUD1*), thereby establishing a negative feedback control on termination [138,198,201–203].

How external signals are conveyed to activate *NRD1* or NRD-counteracting elongation factor genes such as *PAF1*, are only now being dissected, adding a fascinating new layer to gene regulation [20,204]. Moreover, feedback loops between NRD, histone -ubiquitylation, -methylation and -acetylation appear to provide a kinetic control-step prior to Pol II engaging in processive elongation on some genes. This may be critical for the transcriptional licensing of a particular gene [27,29,32,33]. With the growing understanding of transcriptional surveillance mechanisms in humans, evidence is accumulating that yeast is not alone in employing non-coding transcription to fine tune transcription by either antisense transcription, transcriptional interference or transcriptional repression [26,38,44]. Furthermore, if Pol II encounters obstacles during transcription of an open reading frame, elongation factors, such as TFIIS can act to reinstall processivity (see Svejstrup *et al.* in this issue) [205]. If their action is unsuccessful, stalled polymerases are removed and RNAs degraded, in many cases probably involving the exonuclease Rat1 [41,206].

Finally, when reaching a PAS, some Pol II fails to terminate transcription, especially at highly transcribed genes, where up to 60% Pol II complexes may read through the normal transcript end [182,207]. These polymerases are eventually terminated. In many cases such termination events also appear to be coupled to exosome degradation and will lead to complete degradation of the transcript if exosome progression is not inhibited by mRNA packaging factors or competitive re-polyadenylation by Pap1 [109,182,207–209]. How do these escaped transcripts terminate? From the examples described to date, it appears that these polymerases are terminated opportunistically employing any of the other termination modes available. Similar to snoRNA mediated termination, Rnt1 can provide 3′ end mediated termination, but in many more cases, NRD termination will step in, ensuring the immediate degradation of the improperly terminated transcript [182,207,210]. Even though a single Nab3 binding site may elicit termination, Nrd1/Nab3 sites are not recognized within gene bodies more than 1000nt away from the promoter [133,173]. Indeed, when present closer to the 3′ end of a gene, transcript cleavage occurs most likely through recognition of cryptic poly(A) sites within the Nrd1/Nab3-binding site sequence context and will therefore result in transcript stabilization through polyadenylation [173,202,211]. However, past the poly(A) signal this situation changes and Pol II becomes susceptible to Nrd1/Nab3 sites again, ensuing the fail-safe termination of Pol II [182,209,210]. This transcription phase dependent sensitivity of Pol II may be explained by a complex read-out of CTD phosphorylation status (Fig. 1B) that involves competition between Ser5P and Ser2P acting against the inhibitory Tyr1P; About 800 nt downstream of a TSS Tyr1P levels increase, blocking Nrd1 association. However, at or beyond a TTS, this effect is then sharply reduced [59]. Similarly within ORFs, histone modifications that are associated with processive elongation and help to prevent unscheduled initiation (Fig. 1C), also compete with the recruitment of APT to help NRD-dependent termination [29,30]. The opposite scenario can be observed when Pol II fails to recognize a snoRNA terminator and will in many cases continue to transcribe until encountering the next PAS [8,92,138,160,178,212]. Overall it is apparent that yeast Pol II displays a flexible termination process and opportunistically switches its termination mode, depending on the plethora of co-transcriptional cues it receives.

## 6 Co-transcriptional control

**Fig. 3:**
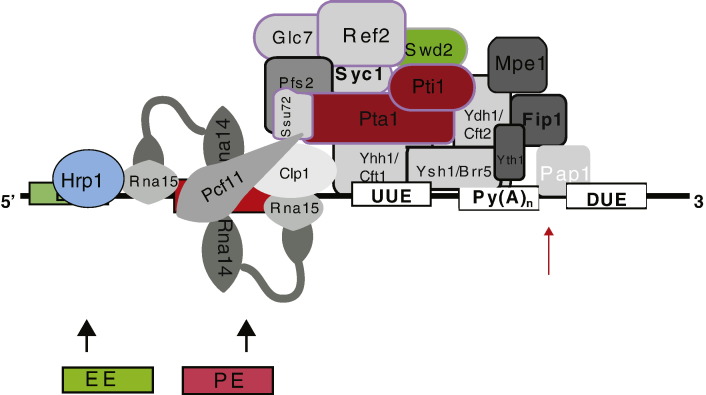
Posttranslational modifications of 3′ end processing factors. A) Component factors of CPF/CF or NRD that are subject to posttranscriptional modifications and that can influence termination efficiency and choice (red phosphorylation, green ubiquitination, blue methylation). Phosphorylation: Pti1 is possibly phosphorylated and activated as a polyadenylation suppressor in situations that activate nuclear surveillance [229]. Pta1 is, when phosphorylated unable to support polyadenylation [213]. Ubiquitination: Swd2 ubiquitination feeds back on H3K4 di- and tri-methylation establishing a kinetic feedback loop [32,33]. Methylation: Hrp1 methylation is required for its nuclear export in an intricate network connecting elongation rate, termination and mRNA export involving Npl3 and the THO complex [56,97,230].

With all these obstacles to productive transcription it appears likely that organisms have evolved additional means to ensure sufficient transcriptional activity on the one hand and to allow for rapid response to external stimuli on the other. As mentioned above, NRD activity is modulated in response to external stimuli [20]. One such modulation appears to be brought about by Nrd1 phosphorylation that is also influenced by the expression levels of Nab3 [20,186]. Similarly, extensive posttranscriptional modifications of the CPF/polyadenylation pathway have been described in mammalian cells, but are also described for their yeast counterparts (Fig. 3) [32,33,213–215]. These modifications not only influence termination efficiency, but also termination position and PAS choice [216]. Based on deep-sequence analysis of Pol II transcript 3′ ends, more than half of all yeast and human transcripts can be terminated at alternative poly(A) signals [50,217]. Initial observations suggest that there appears to be a trend from usage of proximal poly(A) signals in developing, fast proliferating cells to distal poly(A) signals in differentiated, resting cells [216]. In mammals the latter will contain longer 3′ UTRs that often harbour miRNA cognate sequences and are therefore more amenable to cytoplasmic translational or RNA stability control. Alternative PAS seem to be evolutionarily plastic allowing for tissue and species specific adaptations, just as Nrd1/Nab3 binding sites appear to be depleted from yeast sense coding sequences (Porrua-Fuerte *et al.* 2012 *in press*) [216]. It would be surprising if the future exploration of yeast 3′ UTRs doesn't also reveal mechanisms by which mRNA expression is similarly regulated.

On the other hand, PAS choice also feeds back to transcriptional levels; a strong PAS correlates with higher levels of PIC formation and for many genes with the physical association of TSS and TTS, forming a structure referred to as a gene loop [23,218,1]. The phenomenon of gene looping relates to the dynamic interaction of promoter and terminator regions that in effect generates a circular chromatin conformation across the gene [219,220]. These structures are transcription dependent and can act to enhance transcriptional memory of a gene's previous transcriptional state [220,221]. Recently gene loops have also been shown to promote transcriptional directionality on a gene. They act to favour mRNA synthesis over promoter associated antisense ncRNA synthesis [222]. Another example of RNA processing affecting expression levels is the accurate definition of first exons that is also reflected in histone modifications [223] Nucleosome positioning, gene looping and histone modifications may all combine to help transmission of the transcriptional status of a given gene to following polymerases, in effect providing a type of transcriptional memory.

Fast growing cells exploit this connection by over-expressing polyadenylation factors and selectively express RNAs with short 3′UTRs [224,225]. In the case of the human Rtt103 termination factor homologue CREPT, efficient 3′ end formation aids high expression of proliferation stimulating genes by establishing gene loop dependent transcriptional memory [226]. However excessive levels of transcription often result in DNA damage as detailed by Aguilera and colleagues in this issue. Consistent with this the human “polyAosome” co-purifies with DNA-damage repair factors and yeast Rtt103 specifically localises to DNA breaks. This may provide a way to ensure timely termination of polymerases that are prone to run into a DNA break [227].

In the light of the research summarized here, we argue that transcription termination constitutes an important layer in the control of gene expression. We anticipate that revelations from future research will uncover even more intricate interweaving between transcription termination and chromatin associated gene expression.
